# Reticulocyte hemoglobin equivalent to detect thalassemia and thalassemic hemoglobin variants

**DOI:** 10.1111/j.1751-553X.2012.01442.x

**Published:** 2012-07-06

**Authors:** Å A Sudmann, A Piehler, P Urdal

**Affiliations:** * Department of Medical Biochemistry, Oslo University Hospital UllevålOslo, Norway; † Fürst Medical LaboratoryOslo, Norway

**Keywords:** Thalassemia, hemoglobinopathy, reticulocytes, hemoglobin, RBC, ferritin

## Abstract

**Introduction:**

Thalassemia and iron deficiency may both result in hypochromic microcytic anemia. Hematological algorithms that differentiate the two are mainly established in adult selected diagnostic groups. We aimed at creating an algorithm applicable in the presence of children, hemoglobin variants, and iron deficiency.

**Methods:**

Our study material constituted blood samples referred during 1 year for routine diagnostics of hemoglobinopathy. We included 443 samples, of which 37% were from children 3 months or older. We found β-thalassemia trait (*n* = 100), α-thalassemia (*n* = 75), combined α-/β-thalassemia (*n* = 14), hemoglobin variants (*n* = 42), and no-hemoglobinopathy (*n* = 207), of whom 107 had a ferritin at or below 20 μg/L. We included reticulocyte hemoglobin equivalent, ferritin, and erythrocyte count in our algorithm.

**Results:**

Our algorithm differentiated β-thalassemia trait from no-hemoglobinopathy with a sensitivity of 99% at 83% specificity. It performed better than other published algorithms when applied to all patient samples, while equally or moderately better in the 63% adult samples. Our algorithm also detected the clinically significant α-thalassemias, and most of the combined α-/β-thalassemias and thalassemic hemoglobin variants.

**Conclusion:**

Our algorithm efficiently differentiated thalassemia and thalassemic hemoglobin variants from iron deficiency in children and adults.

## Introduction

Hemoglobinopathies, comprising thalassemias and hemoglobin variants, affect millions of people worldwide and especially populations in the Mediterranean, African and Asian areas [1–3]. Coinciding, about one-fourth of the world's women and children are iron deficient [4]. Thus, reliable and efficient diagnostic ways to distinguish between thalassemic and iron restricted hypochromic microcytic anemia are desirable.

Several algorithms based on erythrocyte indices have been proposed to differentiate β-thalassemia trait (BTT) from iron deficiency anemia (IDA) [5–10]. These algorithms are mainly established in adult populations free of coexisting α-thalassemias, hemoglobin variants, and other types of anemia.

Measurement of hemoglobin in reticulocytes has proved useful for the diagnosis and follow-up of IDA [11], while its use in hemoglobinopathies has been less extensively elaborated [12–16]. Reticulocyte hemoglobin is available on both Advia (reticulocyte hemoglobin content; CHr) [17–19] and on Sysmex (reticulocyte hemoglobin equivalent; Ret-He, formerly Ret-Y) [20–24]. The two reticulocyte hemoglobin methods produce essentially similar results [25].

The purpose of our study was to explore the diagnostic value of Ret-He, erythrocyte mean corpuscular hemoglobin (MCH), or volume (MCV) together with ferritin and erythrocyte count in identifying thalassemias and thalassemic hemoglobin variants in children and adults in an unselected 1-year regional hospital routine production.

## Materials and Methods

### Study samples

The Department of Medical Biochemistry at Oslo University Hospital Ullevaal is the major laboratory performing hemoglobinopathy diagnostics in Norway. Five hundred and eighty-four EDTA full blood samples from patients 3 months or older referred to our laboratory for a hemoglobinopathy evaluation between 25 February 2009 and 24 February 2010 were eligible for inclusion. These constituted samples from inpatients (25%) and outpatients (28%) at our hospital, from other hospitals (12%), and general practitioners (35%). The main reasons for referral were anemia and/or microcytosis. Based on the following criteria, we excluded 141 samples from our study: known hemoglobinopathy (*n* = 99), previously performed, normal hemoglobin high pressure liquid chromatography (HPLC) analysis (*n* = 7), subsequent samples from patients already included in our study (*n* = 3), pregnancy with an unknown gestational age (*n* = 4) or postpartum (*n* = 1), suspected or confirmed blood transfusion within the last 3 months (*n* = 7), samples requested from relatives of patients included in the study (*n* = 2), lacking measurements of Ret-He (*n* = 2) or ferritin (*n* = 8), and lacking α-globin gene deletion analysis (*n* = 7) or sequencing of the β-globin gene (*n* = 1). In total, 443 samples were included in the study. Of these, 162 (37%) were from children below the age of 18.

### Methods

Sample evaluation included an EDTA full blood count (FBC, Sysmex XE-2100; Sysmex, Kobe, Japan), hemoglobin HPLC analysis (Variant; Bio-Rad, Hercules, CA, USA), and measurement of serum or plasma ferritin (chemiluminescence immunoassay; Advia Centaur, Siemens, Tarrytown, NY, USA). In samples of known sample age (89%), FBC was performed within 43 h (median) after sampling.

We used HPLC to identify hemoglobin variants, to exclude BTT in children aged 3–6 months when HbA_2_ < 3.0% and to diagnose BTT in those aged >6 months when HbA_2_ > 3.6%. Samples from patients below the age of 3 months were not eligible for inclusion, as HbA_2_ is not fully developed at this age and, therefore, cannot be used to rule out BTT [26]. When HbA_2_ was in the range 3.2–3.6% and a β^+^-thalassemia was suspected, sequencing of the β-globin gene was performed by the Department of Medical Genetics (DMG) at Oslo University Hospital Ullevaal, Norway (*n* = 2). In this study, we included both β^+^-thalassemia and β^0^-thalassemia in the BTT diagnostic group. When considered clinically significant, hemoglobin variants and β-thalassemia were confirmed by sequencing of the β-globin gene by the DMG (*n* = 14).

When either a β- or a δβ-globin gene deletion was suspected, gene analysis was performed. In the first part of our study period, multiplex ligation-dependent probe amplification (MLPA) using the SALSA MLPA kit P102 HBB (MRC-Holland, Amsterdam, the Netherlands) was performed by the DMG (*n* = 2). From 16 September 2009, we performed copy number variation (CNV) analysis in our own department (*n* = 4). For this, real-time polymerase chain reaction (PCR) was performed on an ABI 7900HT (Applied Biosystems, Foster City, CA, USA) using TaqMan probes flanking the β-globin gene (TaqMan® Gene Specific Assays; Hs04398628_cn and Hs06278819_cn; Applied Biosystems) and a TaqMan® CopyNumber Reference Assay, RNase P (cat#4403328; Applied Biosystems) [27]. TaqMan probes were designed to detect all β- and δβ-globin gene deletions published in HbVar (http://globin.cse.psu.edu/hbvar/menu.html).

If either MCH or MCV was below the lower sex- and age-specific reference limit, we also tested for the seven α-globin gene deletions -α^3.7^, -α^4.2^, --^SEA^, --^FIL^, --^THAI^, --^MED^ and -(α^20.5^) using our multiplex gap-PCR assay [28] (*n* = 300). In some samples (*n* = 26) with a low MCH and/or MCV and a normal hemoglobin HPLC, the α-globin gene deletions test was not performed. In the majority of these samples, the hematological aberrations were considered the result of a prior iron deficiency currently being treated. These 26 cases were categorized as no-hemoglobinopathy.

Samples with normal hematology and normal hemoglobin HPLC were classified as no-hemoglobinopathy without any gene test being performed.

The hematological findings in the patients were evaluated against their individual sex- and age-specific reference limits [29–31] as presented in Table 1.

**Table 1:** Sex- and age-specific reference limits* for erythrocyte count, Ret-He, MCH, and MCV

	Upper ref. limit		Lower ref. limit					
		
	RBC (10^12^/L)		Ret-He (pg)		MCH (pg)		MCV (fL)	
				
Age group	M	F	M	F	M	F	M	F
90–180 days	4.8	4.8	23	24	24	24	74	75
0.5–<2 years	5.1	5.0	23	24	23	23	70	71
2–<6 years	5.0	4.9	25	26	24	24	71	72
6–<12 years	5.0	5.0	24	25	25	25	74	76
12–<18 years	5.3	4.9	27	28	25	25	77	77
>= 18 years	5.7	5.2	30†	27†	27	27	82	82
Pregnant 1. trimester	–	4.6	–	30‡	–	30	–	84§
Pregnant 2. trimester	–	4.5	–	30‡	–	30	–	85§
Pregnant 3. trimester	–	4.4	–	29‡	–	29	–	84§

RBC, erythrocyte count; Ret-He, reticulocyte hemoglobin equivalent; MCH, mean corpuscular hemoglobin; MCV, mean corpuscular volume; M, male; F, female.

* Three months to 17 years [29], 18 years or older [30], and pregnant [31]. Upper ref. limit, upper reference limit; Lower ref. limit, lower reference limit.

† Reference [29].

‡ Chosen as identical to pregnant MCH lower reference limit since pregnant Ret-He lower reference limits are not established.

§ Reference [30] MCV lower reference limit corrected for changes during pregnancy [31].

### Statistical analysis

We performed receiver operating characteristic (ROC)-curve analysis by using GraphPad Prism^©^ 5.04 (GraphPad Software Inc., San Diego, CA, USA).

### Ethics

Our study was approved by the Regional Committee for Medical Research Ethics South-East Norway.

## Results

### Diagnosis, sex-, and age distribution

A hemoglobinopathy diagnosis was assigned to 53% (236 of 443) of the study samples (Table 2). BTT was the largest group, but α-thalassemias and hemoglobin variants occurred frequently as well. Five samples had hematological aberrations typical of thalassemia, but normal hemoglobin HPLC and genetic testing. These samples were excluded from the remaining figures and tables, as they could not be allocated to either of the diagnostic groups. No hemogobinopathy was detected in 47% (207 of 443) of the samples. Absence of α-thalassemia was confirmed with gene analysis in 38% (78 of 207) of these. In our material, samples from adult females constituted 42%, adult males 21%, and children below the age of eighteen 37%. This age distribution was essentially similar in the diagnostic groups except in BTT where adult males outnumbered adult females.

**Table 2:** Mean values of the hematological findings in the no-hemoglobinopathy and hemoglobinopathy groups

		*n*	%	RBC (10^12^/L)	Ret-He (pg)	MCH (pg)	MCV (fL)	Ferritin (μg/L)	Hb (g/L)
No-hemoglobinopathy									
Ferritin <5		30	8	4.3	18	19	67	2	81
Ferritin 5–20		77	17	4.5	26	25	80	11	111
Ferritin >20		100	23	4.3	29	27	86	159	117
Total		207	47	4.4	27	25	81	83	110
β-thalassemia trait (BTT)									
Ferritin <5		0	0	–	–	–	–	–	–
Ferritin 5–20		16	4	5.8	19	18	60	11	105
Ferritin >20		84	19	5.8	20	19	63	129	112
Total*		100	23	5.8	20	19	62	110	111
α-thalassemia									
-α^3.7^/αα -α^4.2^/αα		36†	8	5.0	24	22	73	60	112
-α^3.7^/-α^3.7^		15‡	3	5.4	23	22	71	52	118
--^SEA^/αα		24§	5	5.9	22	21	67	191	122
--^MED^/αα									
--^FIL^/αα									
Total		75	17	5.4	23	22	71	100	116
Combined α-/β-thalassemia	Total¶	14	3	5.8	21	20	67	71	118
Suspected thalassemia	Total**	5	1	5.9	21	19	63	70	113
Hemoglobin variant	Total††	42	9	4.9	25	24	72	83	116
Total	Total	443	100	5.0	24	23	73	91	112

RBC, Ret-He, MCH, and MCV are explained in Table 1; Hb, hemoglobin.

* Includes 1 compound HbD and 1 δβ-thalassemia.

† Includes 3 -α^4.2^/αα and 33 -α^3.7^/αα (including 3 compound HbD and 1 compound HbS).

‡ Includes 2 compound HbS and 1 compound HbC.

§ Includes 19 --^SEA^/αα, 4 --^MED^/αα and 1 --^FIL^/αα.

¶ Includes 9 combined -α^3.7^/αα/BTT and 5 combined -α^3.7^/-α^3.7^/BTT.

** These samples showed typical hematological aberrations, but normal hemoglobin HPLC and genetic testing.

†† Includes 22 HbE (14 heterozygous including 1 compound -α^3.7^/αα, and 8 homozygous including 1 compound -α^3.7^/αα), 12 HbS (9 heterozygous including 1 compound β-thalassemia, and 3 homozygous), 4 HbC, and 4 HbD.

### Hematology

Table 2 shows the mean of various hematological parameters in no-hemoglobinopathy and in different groups of hemoglobinopathies. All the thalassemias and thalassemic hemoglobin variants showed low mean Ret-He, MCH, and MCV, as did the no-hemoglobinopathies with ferritin at or below 20 μg/L. The mean erythrocyte count was increased in BTT, heterozygous α^0^-thalassemia, combined α/β-thalassemia, and in the group of suspected thalassemia.

### Iron deficiency

Ret-He, MCH, and MCV diminished with decreasing ferritin both in the no-hemoglobinopathy group and in BTT (Table 2). Iron deficiency (ID), defined as ferritin at or below 20 μg/L, was frequent in the no-hemoglobinopathy group, whereas less frequent in the other diagnostic groups. Severe ID, defined as ferritin below 5 μg/L, was present in 14% of no-hemoglobinopathies, 5% of hemoglobin variants, and 4% of α-thalassemias, while absent in BTT and in combined α/β-thalassemia. Far more adult females (14%) than adult males (1%) had a severe ID.

### Ret-He and erythrocyte count

**Figure 1:**
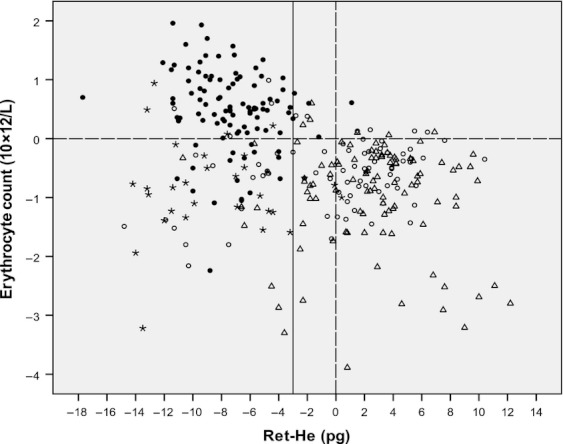
Ret-He and erythrocyte count in BTT and in no-hemoglobinopathy. The dashed lines represent the sex- and age-specific upper erythrocyte count reference limit (horizontal line) and lower Ret-He reference limit (vertical line). The solid vertical line is drawn at three pg below sex- and age-specific lower Ret-He reference limit. BTT, • (*n* = 100). No-hemoglobinopathy samples are subdivided in three groups according to ferritin concentration as * (Below 5 μg/L, *n* = 30), ○ (5–20 μg/L, *n* = 77) and △ (Higher than 20 μg/L, *n* = 100).

All cases of BTT showed either a decreased Ret-He and/or an increased erythrocyte count (Figure 1). Ret-He was below the lower reference limit in 99% of cases and three pg or more below the lower reference limit in 96% of cases. Erythrocyte count was above the upper reference limit in 83% of the BTT cases.

In the no-hemoglobinopathy group, a low Ret-He related closely to severe ID: A ferritin level below 5 μg/L was found in 27 of 57 patients with a Ret-He three pg or more below the lower reference limit, while in only 3 of 150 among those above that cut off.

A highly similar distribution to the one shown in Figure 1 was found when erythrocyte count was plotted against MCH or MCV instead of Ret-He (data not shown).

### ROC-curve analysis

We performed ROC-curve analysis to evaluate the ability of Ret-He, MCH, and MCV as single parameters to discriminate between BTT and no-hemoglobinopathy. At near 100% sensitivity, the specificity of the parameters varied between 64 and 71%. By lowering the sensitivity cut off to 97%, the specificities increased to 69–75%.

### Algorithm to detect thalassemia and thalassemic hemoglobin variants

Based on the results of Figure 1 and of the ROC-curve analysis, we have evaluated the following algorithm for discriminating between BTT and no-hemoglobinopathy. BTT is to be suspected if:

- Ret-He is 3.0 pg or more below the lower reference limit and ferritin is above 4 μg/L.
- And/or Ret-He is below the lower reference limit and the erythrocyte count is above the upper reference limit.

We also evaluated similar algorithms based on MCH or MCV instead of Ret-He. In doing this, we subtracted 3.0 pg and 3.0 fL from the MCH and MCV lower reference limit, respectively.

We compared our algorithm to a selection of previously published algorithms as summarized in Table 3 [5–10]. The different algorithms were compared at 99–100% sensitivities (false-negative cases of BTT = 0–1%) as it is desirable to keep the number of missed BTT cases at a minimum. At these sensitivities, our algorithm with Ret-He, MCH, or MCV yielded specificities of 83–85% (false-positive cases of BTT = 15–17%), whereas the specificities of the other algorithms varied between 52 and 80% (Table 3). As the previously published algorithms were developed primarily in adults, we also compared all the algorithms in adults only. At 100% sensitivity (false-negative cases of BTT = 0%), the specificities of our algorithm with Ret-He, MCH, or MCV remained essentially unchanged at 81–84% (false-positive cases of BTT = 16–19%), whereas the specificity of the other algorithms improved to 61–87% (Table 3). Our results were essentially unchanged when our algorithm was based on the Ret-He reference limits proposed by Canals *et al*. [14] instead of Soldin *et al*. [29] (results not shown).

**Table 3:** Specificity (%) of different algorithms in discriminating between BTT and no-hemoglobinopathy given a BTT detection sensitivity of 99% (all ages) or 100% (age >17 years)

			All ages (*n* = 307)		Age >17 years (*n* = 193)	
				
Algorithm	Parameters	Cut off	Specificity	AUC	Specificity	AUC
This study	Ret-He	–	83	–	81	–
	MCH	–	83	–	81	–
	MCV	–	85*	–	84	–
Ehsani [10]	MCV–10xRBC	<26	80	0.96	87	0.98
Shine and Lal [8]	MCV^2^ × MCH/100	<1243	79	0.90	83	0.92
Sirdah [9]	MCV–RBC–3×Hb	<38	71	0.96	82	0.98
Mentzer [6]	MCV/RBC	<17	70	0.95	78	0.98
Srivastava [7]	MCH/RBC	<6	57	0.93	61	0.96
England & Fraser [5]	MCV–RBC–5×Hb	<20	52	0.92	65	0.94

AUC, area under curve.

* Sensitivity = 100%.

In our material, both α-thalassemias and hemoglobin variants occurred frequently (Table 2). Therefore, we evaluated the sensitivity of the various algorithms in identifying these conditions (Table 4) using essentially the same cut off values as in Table 3. When all ages were grouped together, all algorithms correctly identified all heterozygous α^0^-thalassemias, and a variable majority of combined α-/β-thalassemias, homozygous α^+^-thalassemias, and HbE. Our algorithm yielded no false-negative cases of heterozygous α^0^-thalassemias, whereas 7–21% in combined α-/β-thalassemias, 0–13% in homozygous α^+^-thalassemias, and 18–45% in HbE. In adults, all algorithms correctly identified all heterozygous α^0^-thalassemias and combined α-/β-thalassemias, most homozygous α^+^-thalassemias, and the majority of HbE. The Srivastava index [7] identified most heterozygous α^+^-thalassemias for a specificity of 61% (results not shown). In adults, our algorithm yielded no false-negative cases of heterozygous α^0^-thalassemias nor combined α-/β-thalassemias, whereas 0–8% in homozygous α^+^-thalassemias and 13–44% in HbE.

**Table 4:** Sensitivities (%) of different algorithms in detecting hemoglobinopathies other than BTT

	All ages					Age >17 years				
		
Algorithm*	-α^3.7^/αα -α^4.2^/αα (*n* = 36)	-(α^3.7^)_2_† (*n* = 15)	-^SEA^/αα -^MED^/αα -^FIL^/αα (*n* = 24)	αβ-thal.‡ (*n* = 14)	HbE (*n* = 22)	-α^3.7^/αα -α^4.2^/αα (*n* = 20)	-(α^3.7^)_2_† (*n* = 12)	-^SEA^/αα -^MED^/αα -^FIL^/αα (*n* = 19)	αβ-thal.‡ (*n* = 7)	HbE (*n* = 16)
Ret-He§	47	87	100	86	59	50	92	100	100	63
MCH§	64	93	100	93	55	60	100	100	100	56
MCV§	56	100	100	79	82	55	100	100	100	88
Ehsani	58	87	100	100	64	45	92	100	100	56
Shine and Lal	56	80	100	86	59	45	83	100	100	50
Sirdah	61	93	100	100	82	45	92	100	100	75
Mentzer	69	93	100	100	82	55	92	100	100	75
Srivastava	86	100	100	100	91	75	100	100	100	88
England & Fraser	72	93	100	100	91	55	92	100	100	88

References to the different algorithms are given in Table 3.

* Cut off as in Table 3 except for Ehsani (lower than 27) and Shine and Lal (lower than 1249).

† -α^3.7^/-α^3.7^.

‡ Combined α-/β-thalassemia.

§ This study.

## Discussion

We found Ret-He, MCH, or MCV together with ferritin and erythrocyte count to be powerful in identifying thalassemias and thalassemic hemoglobin variants in children and adults in a 1-year study population with a high prevalence of ID. Although such biochemical analysis cannot replace DNA-based diagnosis in hemoglobinopathy, it does represent an important guiding tool for the clinician.

The combined use of Ret-He, ferritin, and erythrocyte count efficiently identified BTT for a high specificity. Our algorithm performed equally well when Ret-He was replaced by either MCH or MCV. This may be advantageous as Ret-He is not yet available in all laboratories. However, Ret-He conveys additional information compared to MCH or MCV as it essentially normalizes within 2 days of effective intravenous iron treatment of an iron-deficient individual [18] and is, therefore, regarded as superior in our study. In fact, we would expect many of the 40 cases of no-hemoglobinopathy with a low Ret-He and ferritin at or below 20 μg/L positioned in the lower left quadrant of Figure 1 to move into the lower right quadrant upon few days of iron treatment, thereby abolishing thalassemia suspicion.

Hematology analyzers differ systematically in analytical levels of MCH, MCV, and RBC [30]. As these differences are relatively small, analyzer-specific reference ranges are mostly considered unnecessary [30]. Thus, we expect our algorithm to function well also when applied to major hematology analyzers other than the Sysmex used in this study.

Our algorithm yielded higher specificity in separating BTT from no-hemoglobinopathy as compared to a selection of published algorithms [5–10]. When we tested the selection of published algorithms on our adult study population, three of them [10, 8, 9] performed equally to ours, while three performed poorer [5–7].

Most other algorithms have a documented utility in adults, only. Our algorithm includes correction for normal variation in Ret-He, MCH, or MVC and erythrocyte count with sex and age. As illustrated by the high number of children screened for hemoglobinopathies in our study (37%), the applicability of our algorithm to all ages is a considerable strength.

In an unselected study population, α-thalassemias and hemoglobin variants will occur nearly as frequently as BTT. To our knowledge, most of the other published algorithms have not been applied to unselected populations to identify hemoglobinopathies other than BTT. We found both our algorithm and the published ones to differentiate well between heterozygous α^0^-thalassemias and no-hemoglobinopathy (Table 4). They also identified most cases of homozygous α^+^-thalassemias and combined α-/β-thalassemias. On the other hand, the detection rates of the algorithms were lower in HbE and even lower in heterozygous α^+^-thalassemias. The latter was to be expected, as heterozygous α^+^-thalassemias often present without hematological aberrations such as microcytosis. The Srivastava index [7] identified more heterozygous α^+^-thalassemias than did any of the other algorithms, but with a low specificity. The numbers of heterozygous α^+^-thalassemias in our material do probably not reflect the true incidence, as our diagnostic process only detects the ones with low MCH or MCV.

Our algorithm differs from most of the published ones in not being structured as a mathematical formula. In our mind, this facilitates its clinical use. Moreover, our algorithm is different in that it includes Ret-He and ferritin. Ferritin is a frequently used marker of ID and is normally measured in patients suspected of having a hemoglobinopathy. We found ID among both thalassemic patients and those with a hemoglobin variant. Severe ID, with a ferritin below 5 μg/L on the other hand, was neither found in BTT nor combined α-/β-thalassemias, and only infrequently in the other hemoglobinopathies. In BTT, this phenomenon is probably due to ineffective erythropoiesis and repression of hepcidin with subsequent increased iron absorption [32]. One may suspect similar mechanisms in other types of thalassemias as well.

Our study population has a high prevalence of nonethnical Norwegians and thereby constitutes a high-risk group. By 2006, the 123.900 immigrant population of Oslo counted with 50% Asian descent and 18% African descent [33]. In this setting, our algorithm yielded high sensitivities of case detection. Our algorithm is likely to function well when applied to a low-risk population as well. We would expect a low number of false positives, as our main criterion for thalassemia suspicion (Ret-He three pg or more below lower reference limit) rarely will be met in healthy persons. It is more difficult to predict what numbers of false negatives to expect. It is very unusual, though, to find a β- or δβ-thalassaemia carrier with a normal MCH and MCV value except in the presence of coexistent α-thalassemia trait [2]. Heterozygous α^+^-thalassemias, on the contrary, often have normal hematology and would be categorized as false negatives if our algorithm was expected to identify also these patients. In conclusion, we expect our algorithm to identify thalassemic hemoglobin variants and thalassemias apart from heterozygous α^+^-thalassemias, also in a low-risk population.
